# Effects of pesticide-adjuvant combinations used in almond orchards on olfactory responses to social signals in honey bees (*Apis mellifera*)

**DOI:** 10.1038/s41598-023-41818-7

**Published:** 2023-09-20

**Authors:** Wen-Yen Wu, Ling-Hsiu Liao, Chia-Hua Lin, Reed M. Johnson, May R. Berenbaum

**Affiliations:** 1 Department of Entomology, University of Illinois Urbana-Champaign, 505 S. Goodwin Avenue, Urbana, IL 61801 USA; 2 Department of Entomology, Rothenbuhler Honey Bee Research Laboratory, The Ohio State University, 2501 Carmack Road, Columbus, OH 43210 USA

**Keywords:** Agroecology, Chemical ecology, Olfactory system, Extracellular recording

## Abstract

Exposure to agrochemical sprays containing pesticides and tank-mix adjuvants has been implicated in post-bloom mortality, particularly of brood, in honey bee colonies brought into California almond orchards for pollination. Although adjuvants are generally considered to be biologically inert, some adjuvants have exhibited toxicity and sublethal effects, including decreasing survival rates of next-generation queens. Honey bees have a highly developed olfactory system to detect and discriminate among social signals. To investigate the impact of pesticide-adjuvant combinations on honey bee signal perception, we performed electroantennography assays to assess alterations in their olfactory responsiveness to the brood ester pheromone (BEP), the volatile larval pheromone β-ocimene, and the alarm pheromone 2-heptanone. These assays aimed to uncover potential mechanisms underlying changes in social behaviors and reduced brood survival after pesticide exposure. We found that combining the adjuvant Dyne-Amic with the fungicide Tilt (propiconazole) and the insecticide Altacor (chlorantraniliprole) synergistically enhanced olfactory responses to three concentrations of BEP and as well exerted dampening and compensatory effects on responses to 2-heptanone and β-ocimene, respectively. In contrast, exposure to adjuvant alone or the combination of fungicide and insecticide had no effect on olfactory responses to BEP at most concentrations but altered responses to β-ocimene and 2-heptanone. Exposure to Dyne-Amic, Altacor, and Tilt increased BEP signal amplitude, indicating potential changes in olfactory receptor sensitivity or sensilla permeability to odorants. Given that, in a previous study, next-generation queens raised by nurses exposed to the same treated pollen experienced reduced survival, these new findings highlight the potential disruption of social signaling in honey bees and its implications for colony reproductive success.

## Introduction

Among the multiple stresses affecting honey bee health in contemporary apiculture, pesticide residues within hives and in agricultural landscapes where bees forage have been identified as significant contributors to bee mortality and sublethal impacts^1–3^. Formulated pesticides are often more toxic to insects, including non-target pollinators, than are the active ingredients of pesticides^4–7^, suggesting that chemicals other than active ingredients in formulated pesticides and tank-mixes may play a role in the mortality of pollinators and other non-target insects in agroecosystems.

Adjuvants, which are "inert components" in pesticide formulations or tank mixes of agrochemicals, are used to enhance the performance of the active ingredients of pesticides by increasing solubility, dispersion, attachment, residual activity, persistence, and stability^8^. Because they are considered biologically inert, most adjuvants are not subject to the same level of rigorous safety testing as are the insecticidal ingredients. As a result, the toxicity of adjuvants, alone or in combination with pesticides, is not well understood. Adjuvant residues have been detected at high levels (up to 40.5 mg/kg) in North American beehives^9^, as well as on almond flowers^10^ and beebread^11^. Previous studies have shown that adjuvants can inadvertently increase off-target effects of pesticide active-ingredients on animals^12,13^, including honey bees and other pollinators^5,8,14–17^. Despite the toxicity risks, almond pesticide application data from the California Pesticide Information Portal (CalPIP; https://calpip.cdpr.ca.gov/) show that the use of adjuvants, with other pesticides, is common in almond orchards before and during bloom, when two-thirds of U.S. bee colonies are present in almond orchards to provide pollination service. The combined application of pesticides and adjuvants during bloom aims to control pests such as the peach twig borer, as well as fungal diseases such as brown rot, anthracnose, leaf blight, and scab. Commercial spray adjuvants, especially silicone surfactants, can have lethal and sub-lethal effects on honey bees^5,15,17^. For example, organosilicone surfactant adjuvants can impair olfactory learning^18^ and cause acute toxicity to honey bee adults^19^. Additionally, adjuvants can synergize the toxicity of some pesticides^4,6,20,21^ as well as the virulence of black queen cell virus^22^. Given this previous work, there is a distinct possibility that adjuvant applications in pollinator-dependent crops may harm honey bees and potentially other pollinators.

Honey bees have a sophisticated olfactory system that allows them to perceive and respond to social cues in their hive environment, including those involved in interactions with nestmates^23–25^. Insecticide interference with olfactory-mediated responses has been documented in a wide range of species^26–28^, raising the expectation that exposure to pesticides could also hinder the functionality of the olfactory system in honey bees. Even sublethal doses of pesticides can cause deformation of the antennal sensilla of honey bees^29^ and impair learning and memory^30^, resulting in reduced foraging efficiency and colony productivity ^31,32^.

Colony losses characterized by larval and pupal mortality have frequently been reported after almond pollination, and use of tank-mixed insecticide, fungicide, and adjuvants has been implicated in these losses^33^. Dyne-Amic, the fungicide propiconazole, and the insecticide chlorantraniliprole have been used as tank-mixed pesticides^21^. The adjuvant Dyne-Amic, a blend of methylated vegetable oils combined with organosilicone-based non-ionic surfactants, serves as a surfactant and penetrant in agriculture. It enhances pesticide dispersion on plant surfaces and aids in penetrating the leaf cuticle, leading to improved uptake and distribution. Based on the CALPIP database, Dyne-Amic was the most commonly used adjuvant in almond orchards, reaching almost 244,000 cumulative acres in 2016–2018 during the pollination window (February 15–March 15), approximately 50% greater acreage than the second most commonly used adjuvant (reaching approximately 163,000 cumulative acres). Furthermore, synergistic interactions between the fungicide propiconazole and the insecticide chlorantraniliprole can increase toxicity to honey bee larvae^34^ and adults^35^. The combination of the adjuvant Dyne-Amic with this fungicide-insecticide mix can reduce the LC_50_ ratios of these pesticides on adult honey bees^21^ and lower queen emergence rate^36^.

The mortality experienced by larval and pupal bees, as well as the reduced survival of queens, could conceivably result from the sublethal effects of a tank-mixed combination of adjuvant-fungicide-insecticide to which adult honey bees are exposed. Considering the superorganism structure of honey bees societies, adverse impacts of neurotoxic pesticides on worker bees responsible for nurturing developing brood could include reduced olfaction-mediated responses to nestmates, including larvals signals (reviewed in Table 1), with colony-level consequences. The impacts are particularly significant in specific individuals within the colony, particularly queen-destined larvae^21,36^. These larvae experience exposure to remarkably low pesticide levels^36^ or might not even directly interact with the pesticide stressor^37^. Accordingly, we investigated the impact of a sublethal dose of a tank-mix combination on the olfactory responses of bees. To ensure comparability, we used the same batch of treated pollen as was used in the queen rearing experiments conducted by Ricke et al.^36^, who reported lower survival of next-generation queens that had been raised by nurse bees exposed to the combination of Dyne-Amic, propiconazole, the active ingredient in Tilt, and chlorantraniliprole, the active ingredient of Altacor.

**Table 1:** Olfaction-mediated sublethal adverse effects of pesticides on eusocial pollinators.

Species	**Effects** (By I/F/H/A)^a^	**Group** **of MoA**	**Type of Pesticide**	**Active Ingredients**	**Ref.**
**Honey bee —**					
*Apis mellifera*	**Behavior**				
	I	4 (IRAC)	Neonicotinoids	Imidacloprid	^44^
				Thiamethoxam	^45^
	I + F	4 (IRAC) + 1 (FRAC)	Neonicotinoids + MBC fungicides	Thiamethoxam + "carbendazol [*sic*]"	^45^
	**Olfactory learning**				
	I	1 (IRAC)	Organophosphates	Coumaphos	^46^
		2 (IRAC)	Organochlorines	Endosulfan	^47^
			Phenylpyrazoles	Fipronil	^47,48^
		3 (IRAC)	Pyrethroids	Deltamethrin	^47^
		4 (IRAC)	Butenolides	Flupyradifurone	^49^
			Neonicotinoids	Acetamiprid	^48^
				Imidacloprid	^46,50–54^
				Thiacloprid	^55^
				Thiamethoxam	^48^
	H	9 (HRAC)	Organophosphates	Glyphosate	^54^
	F	11 (FRAC)	QoI fungicides	Pyraclostrobin	^56^
		3 (FRAC)	DMI fungicides	Prochloraz	^47^
		7 (FRAC)	SDHI fungicides	Boscalid	^56^
	A	n/a^b^	Surfactants	Organosilicone	^18^
				Nonionic	^18^
	**Sensory receptors (peripheral)**				
	I	3 (IRAC)	Pyrethroids	Fluvalinate	^57^
				Permethrin	^58^
				Tetramethrin	^58^
		4 (IRAC)	Neonicotinoids	Thiacloprid	^55^
	**Central neuron**				
	I	1 (IRAC)	Organophosphates	Coumaphos-oxon	^59^
		4 (IRAC)	Butenolides	Flupyradifurone	^60^
			Neonicotinoids	Clothianidin	^59^
				Imidacloprid	^51,59^
		6 (IRAC)	Avermectins	Ivermectin	^61^
*Apis mellifera scutellata*	**Olfactory learning**				
	I	4 (IRAC)	Neonicotinoids	Imidacloprid	^62^
**Bumble bee —**					
*Bombus impatiens*	**Olfactory learning**				
	F	1 (FRAC)	MBC fungicides	Thiophanate-methyl	^63^
		M 02 (FRAC)	Inorganic fungicide	Sulfur	^63^
		P 07 (FRAC)	Phosphonate fungicides	Mono- and di-potassium salts of phosphorous acid	^63^
*Bombus terrestris audax*	**Olfactory learning**				
	H	9 (HRAC)	Organophosphates	Glyphosate	^64^
**Stingless bee —**					
*Melipona quadrifasciata*	**Olfactory learning**				
	I	4 (IRAC)	Neonicotinoids	Imidacloprid	^62^

^a^I = Insecticide, F = Fungicide, H = Herbicide, A = Adjuvant; ^b^ n/a: not applicable.

We conducted electroantennogram (EAG) assays to evaluate the tank-mix combination of fungicide, insecticide, and adjuvant in terms of changes induced in olfactory responses to social signals. These signals included the semi-volatile brood ester pheromone, simulated by a mix of 10 fatty acids^38^, and the larval volatile pheromone β-ocimene, including both (E)- and (Z)-β-ocimene. Previous research has demonstrated that bee larvae produce the brood ester pheromone^39,40^ and both forms of β-ocimene^41^, which trigger responses from nurse bees. Our aim was to investigate whether these changes in olfactory responses could serve as a potential mechanism underlying alterations in nursing behavior and other social behaviors within honey bee colonies^24,25^. Additionally, we measured changes in olfactory responses to the alarm pheromone 2-heptanone, which has the potential to function as a repellent pheromone, as an explanatory factor underlying changes in defense behaviors^42^. The behaviors encompass actions such as guard bees utilizing alarm pheromones to tag intruders following an attack or other worker bees using the same alarm pheromone to display heightened vigilance and increased agitation^42,43^. The main purpose of this study thus was to uncover potential mechanisms that contribute to changes in social behaviors in response to pesticides and to provide insights that might be linked to the reported decrease in the survival of next-generation brood and queens of honey bees, as observed in our previous study^36^.

## Results

**Figure 1:**
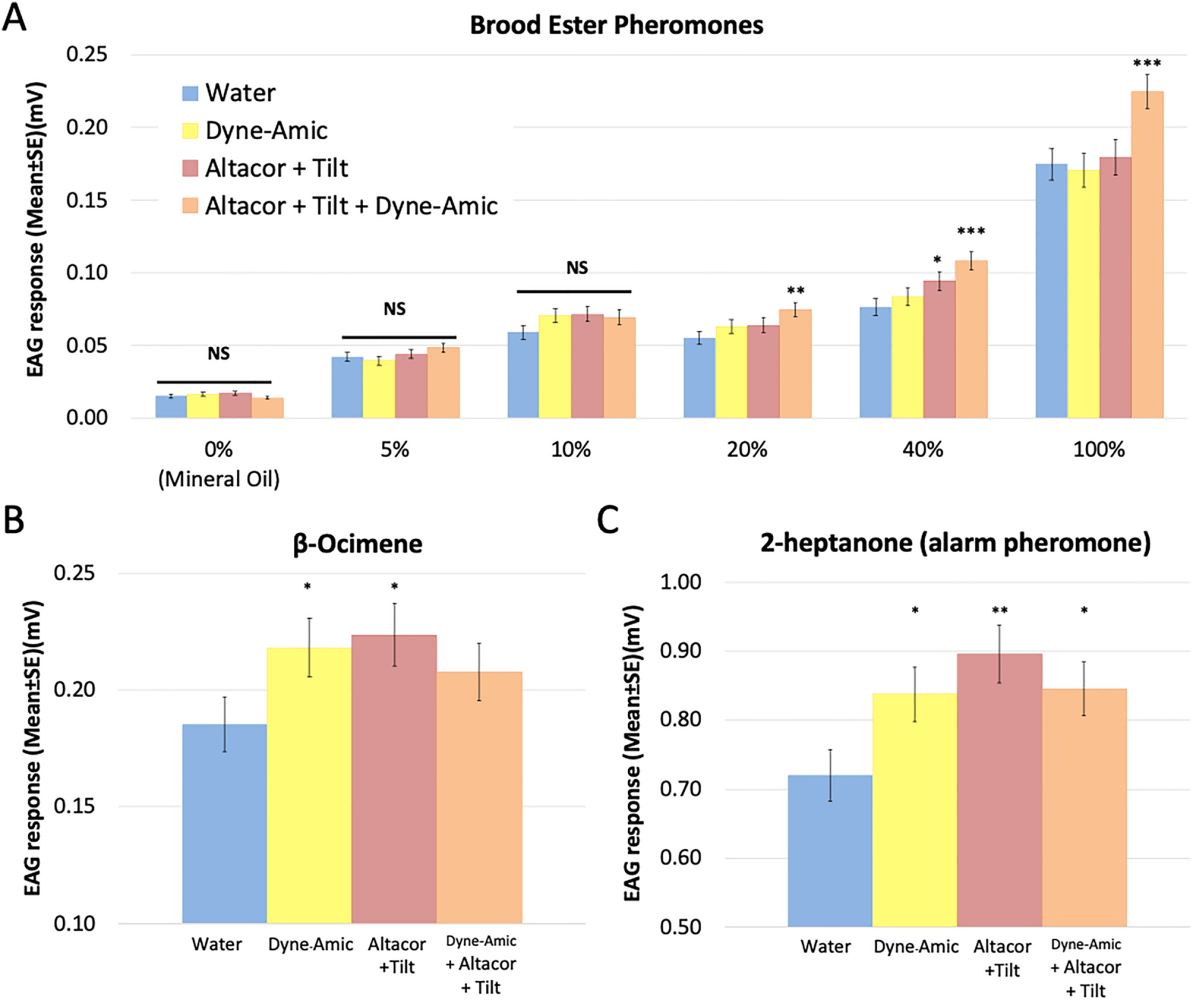
Electroantennogram (EAG) responses of adult honey bee workers to social signals after exposure to the organosilicone adjuvant Dyne-Amic, the fungicide Tilt and the insecticide Altacor, alone or in combination: (**A**) brood-emitted ester pheromone (BEP), (**B**) β-ocimene (larval volatile pheromone), and (**C**) 2-heptanone (alarm pheromone). The EAG responses of bees that consumed pollen with water, representing the control group, are shown in blue. The other three groups, including the Dyne-Amic adjuvant treatment (shown in yellow), the Altacor + Tilt pesticide treatment (shown in red), and the Altacor + Tilt + Dyne-Amic adjuvant-pesticide mixture treatment (shown in orange), are referred to as treatment groups. The asterisk symbol (*) indicates a statistically significant difference in the EAG responses of each treatment group (Dyne-Amic, Altacor + Tilt, or Altacor + Tilt + Dyne-Amic) compared to the EAG responses of bees in the control group that consumed pollen with water, using the same concentration of tested stimuli. The estimated marginal mean of EAG response ± SE is listed. (NS: *p* > 0.05, *: *p* < 0.05, **: *p* < 0.01, ***: *P* < 0.01, N = 106, GEE test).

In general, the olfactory electroantennogram (EAG) responses of honey bees to the 10-in-1 brood-emitted ester pheromone (BEP) showed a dose-dependent pattern (Fig. 1A). However, adjuvant-pesticide-mediated changes in olfactory responses were observed primarily at higher concentrations of BEP. No significant differences were found between any of the treatments and the control at the lowest concentration of BEP stimulus. In addition, in terms of the effects of pesticides on olfactory perception of BEP, exposure to the combination of the fungicide Tilt and the insecticide Altacor enhanced the amplitude of olfactory responses of bees to BEP only at a concentration of 40% (Fig. 1A).

**Figure 2:**
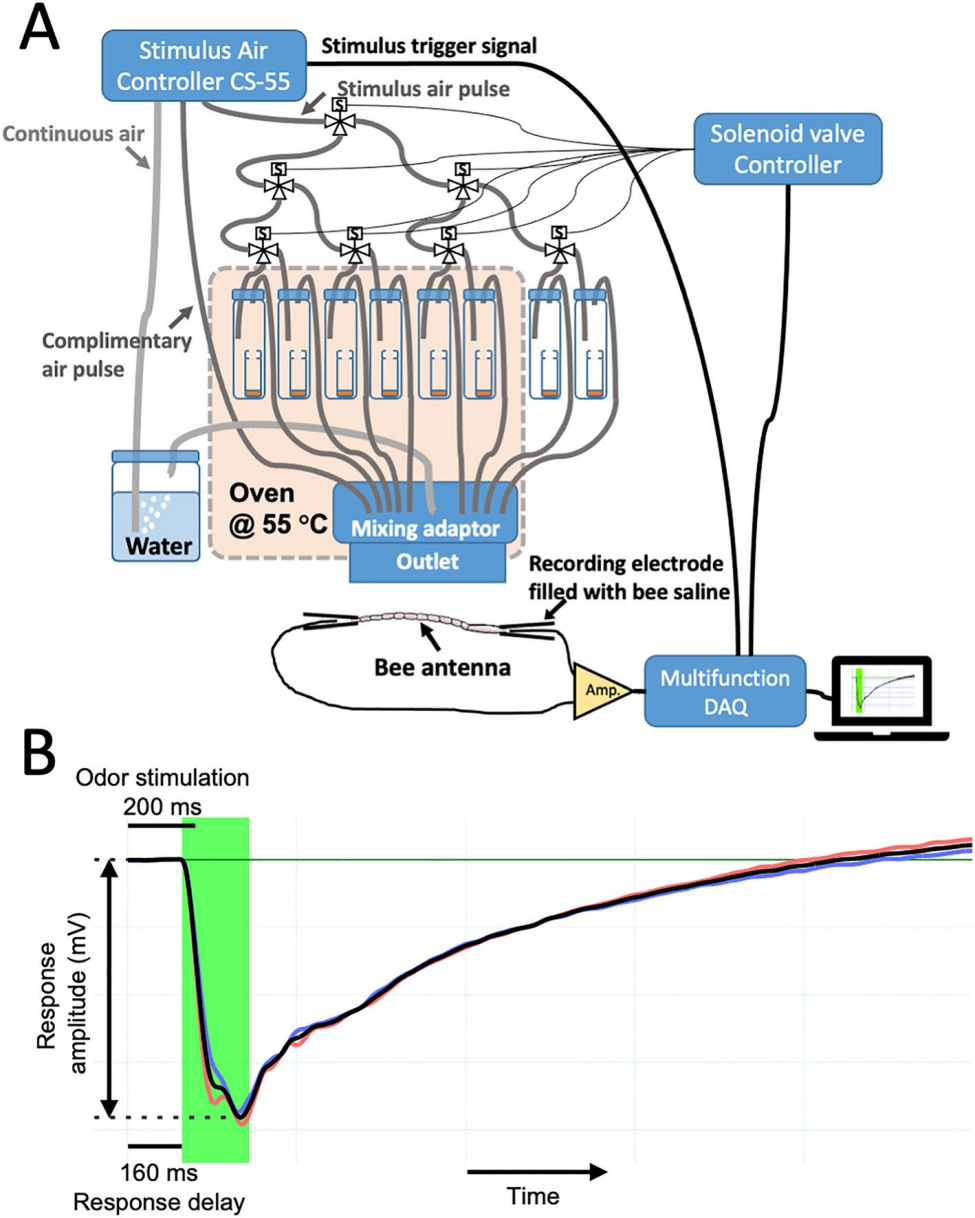
Experimental setup and features of electroantennogram responses. (**A**) Diagram illustrating the overall setup of the electroantennogram (EAG) recording and stimulus delivery apparatus. (This diagram is not drawn to scale. DAQ: data acquisition, Amp: amplifier) (**B**) Features of EAG responses monitored. Duration of odor stimulation is 200 ms. Lag time or response delay is the measured time difference (~ 160 ms) from stimulus introduction to start of voltage drop.

No changes occurred in olfactory responses to BEP when the organosilicone adjuvant Dyne-Amic was administered alone. However, a increased effect was observed in the olfactory responses of bees to 20%, 40% and 100% BEP when the Dyne-Amic was used in combination with Tilt and Altacor. In contrast, Dyne-Amic alone did alter the olfactory response of bees to the larval volatile pheromone β-ocimene (Fig. 1B) and the alarm pheromone 2-heptanone (Fig. 1C) but did not have any significant effects on the responses to the BEP. Exposure of honey bees to Altacor and Tilt resulted in a significant increase in olfactory responses to β-ocimene (Fig. 1B) and 2-heptanone (Fig. 1C), but no synergistic effect on olfactory responses to β-ocimene was observed (Fig. 1B) when the adjuvant Dyne-Amic was used in combination with Tilt and Altacor. Notwithstanding, exposure to Dyne-Amic + Altacor + Tilt still led to heightened sensitivity to 2-heptanone compared to the control group (Fig. 1C). Thus, the effect of the adjuvant-pesticide mixture on β-ocimene indicated a compensatory effect, where the combined influence offset the individual effects of the pesticide or adjuvant alone, resulting in responses similar to the control. On the other hand, for the alarm pheromone, the effect of the adjuvant-pesticide mixture reduced the responses compared to their individual effects, and they were still different from the control, indicating a dampening effect.

## Discussion

The eusocial lifestyle of *A. mellifera* involves a complex series of behavioral and physiological events that affect hive function and reproductive success. Sublethal doses of pesticides can disrupt olfaction-mediated behaviors and olfactory neurotransmission (Table 1), which can affect the coordination of events and result in a reduction in colony survival. In this study, we found that the fungicide Tilt and the insecticide Altacor, with the active ingredients propiconazole and chlorantraniliprole, respectively, together and in combination with the organosilicone surfactant adjuvant Dyne-Amic altered olfactory responses of worker bees to the brood ester pheromone and the alarm pheromone 2-heptanone. This finding suggests that these commonly used pesticides, when applied in a tank mix, may interfere with honey bee communication and reproductive systems that rely on olfactory cues^23–25^.

In addition, we observed complex interacting impacts on honey bee olfactory responses when honey bees were exposed to a combination of pesticides and adjuvants. The effects are not additive (i.e., the sum of the individual effects of pesticides and adjuvants), indicating a more complex relationship among them. The adjuvant Dyne-Amic in combination with a DMI (demethylation inhibitor) fungicide and a diamide insecticide synergistically affected the olfactory responses to the brood ester pheromone, while the combination triggers other modulatory effects on responses, including dampening effects on the alarm pheromone response and compensatory responses to the larval signal β-ocimene. Our results with the BEP are consistent with findings reported in several previous studies. Combinations of pesticides at sublethal levels can lead to additive or synergistic effects on olfactory responses and other aspects^65,66^. Additionally, the organosilicone surfactant adjuvants, such as those present in Dyne-Amic, interfere with the olfactory learning ability of adult honey bees^18^ and can also increase the toxicity of some pesticides^4,6,20,21^. The impaired olfactory responses may be attributed to toxic effects on the peripheral olfactory system of honey bees, central nervous system, or both. Sublethal doses of pesticides can induce neurotoxic effects in honey bees, including altered synaptic transmission, oxidative stress, and apoptosis^31,32,60,67–69^. Furthermore, Asian honey bees (*Apis cerana*) from areas with high pesticide use exhibit impaired olfaction, characterized by decreased proboscis extension response during olfactory learning, alterations in the expression of Calpain 1 (an important calcium-binding protein), and reduced calcium levels in their brains^29^. Therefore, our study suggests that the combination of pesticides and adjuvants can alter honey bee neurons and thereby interfere with olfactory-mediated behaviors.

One of the unexpected findings in our study was the observation that pesticide and adjuvant exposure led to an increase in the amplitude of EAG responses to brood and alarm pheromones. The amplitude of EAG responses is influenced by factors such as the number of receptors, the receptor response of individual olfactory receptor neurons, the density of responsive neurons, and the firing rate of neurons involved in olfaction^70^. The larger EAG responses we observed suggest an enhanced sensitivity or altered selectivity of olfactory receptors in the olfactory system to the tested stimuli. Because adult bees are less likely to exhibit an increase in receptor number, this enhanced sensitivity may be attributed to several mechanisms, e.g., alterations in receptor sensitivity, changes in the expression of receptors, chemosensory or odorant-binding proteins, or modifications in signaling pathways^71^ due to exposure to pesticides or adjuvants. Odorant-binding proteins and chemosensory proteins play a crucial role in modulating the sensitivity of the olfactory system^72^. In *Drosophila*, the absence of odorant-binding proteins in certain sensilla might lead to increased electrophysiological responses^73,74^. Mayack^75^ identified 22 chemicals, including agricultural chemicals and alpha-terpineol, an adjuvant and fungicide, as high-affinity binders of OBP14, an odorant-binding protein of honey bees. These chemicals have all been found in beehives, suggesting that they may affect olfactory perception. Additionally, exposure to neonicotinoid pesticides can alter the expression of odorant-binding proteins and chemosensory proteins in bees^76–78^, potentially changing the sensitivity and perception of odors, including brood pheromone, queen mandibular pheromone, and trail pheromone^78^.

Another possible mechanism is that pesticides and adjuvants may disrupt the structure of antennal sensilla, where olfactory receptor neurons are housed. This disruption can increase sensilla permeability to odorants, potentially enhancing receptor sensitivity to BEP. Evidence from *A. cerana* from pesticide-intensive sites has revealed distortions in sensilla, including crack-like marks on the sensory placode and campaniform structures across the antennal segments^29^. Similarly, in the aphid *Aphis craccivora* and the tick *Haemaphysalis longicornis*, exposure to certain essential oils deformed antennal segments and sensilla^79,80^, indicating impacts of such substances, oil-based adjuvants and pesticides, on olfactory structures. In addition, permeability is also a function of adjuvants, particularly oil-based or surfactant-based ones, as they significantly enhance the absorption of pesticides on plant surfaces^81^ and on insect cuticle^82^. Thus, adjuvants may also facilitate increased penetration of odors and pheromones through the sensilla cuticle, ultimately leading to altered olfactory receptor sensitivity and synergistic effects.

Our results suggest that, at the level of antennal (peripheral) olfactory receptor neurons, the first step in olfactory perception, adjuvants and insecticides altered neural responses. However, the correlated behavioral output of bees in response to these changes is not yet clear. It is important to acknowledge that the response of insects to volatile chemicals or pheromones is not determined by the peripheral olfactory receptor neurons alone. The peripheral information undergoes complex integration and reshaping^83^, involving the combinations and interaction of various stimuli in the antennal lobe and higher brain centers^84–87^. In the moth *Agrotis ipsilon*, Rabhi et al.^88,89^ found that the neonicotinoid clothianidin could either increase or decrease behavioral sex pheromone responses depending on pesticide concentration, but the responses of olfactory receptor neurons remained unchanged, while the sensitivity of intracellularly recorded antennal lobe output neurons was correspondingly altered. These findings suggest the involvement of the antennal lobe in the mediation of clothianidin-induced behavioral changes^89^. Lucas and Renou^90^ also showed that deltamethrin, bioresmethrin, and DDT did not alter the amplitude of the EAG in response to pheromone stimulation, but action potential initiation was sensitive to these insecticides.

As a rule, olfaction involves the integration of signals within the central nervous system, including external cues and internal physiological states of bees^91,92^. While an increase in EAG amplitude was observed, it does not necessarily indicate a corresponding change in behavioral responses, as sensory processing in the brain also plays a role in shaping behavior^70,93^. However, in the parallel study that used the same batch of treated pollen as this study, Ricke et al.^36^ found that queens raised by workers fed with pollen treated with Tilt, Altacor, and the Dyne-Amic exhibited reduced overall survival rates compared to the control group^36^. Our findings suggest that the alterations in EAG responses to brood pheromones in workers may be linked to subsequent nursing quality and reproductive success of brood and the next generation of queen-rearing^36^.

In addition, our findings suggest that pesticide exposure can have varying effects on different sensory stimuli. Even when exposed to the same combination of pesticides and/or adjuvants, the responses of olfactory neurons can be selectively affected, resulting in inconsistent levels of interfering pheromone perception^94,95^. This may be potentially due to factors like receptor saturation or feedback loops, leading to altered behaviors^25,96^. Receptor specificity for different odors may also contribute to the observed diverse responses^25^. This phenomenon of inconsistent interference levels is not limited to honey bees, as Lalouette et al.^97^ observed selective and differential effects of the pyrethroid insecticide deltamethrin on different sensory stimuli in the cotton leafworm *Spodoptera littoralis*. They found that deltamethrin had an impact on male responses to sex pheromones, while their response to host plant odorants remained unaffected. These findings indicate that the effects of pesticides on sensory perception can vary depending on the specific stimuli involved.

Moreover, bees interact and manage colony organization using chemical cues, and almost every aspect of honey bee colony life involves pheromones. In this study, we examined only the alarm and brood pheromones, which are used to recruit bees for colony defense and for larval care, respectively. Other pheromone responses may also be affected by the combination of adjuvant and pesticides. Overall, our data, combined with other studies showing olfaction-mediated communication deficits in bees exposed to adjuvants and insecticides, suggest that commonly used adjuvants, insecticides, and fungicides could be partially responsible for the observed decline in eusocial pollinator populations. Additionally, concurrent exposure to multiple pesticides and adjuvants may increase risks to the health of these indispensable pollinators in agroecosystems.

## Materials and methods

### Experimental bees and their diet treatments

The experiment was conducted with the western honey bees, *Apis mellifera*, kept in an apiary of the University of Illinois Bee Research Facility, Urbana, IL (40.072773, -88.21944). Frames of capped brood were collected from two naturally mated queen hives and incubated in a dark room (34 ± 1 °C, 50 ± 10% RH) to obtain newly emerged workers.

Adult honey bee workers were collected within 24 h after emergence and were randomly introduced into Petri dish cages^98^, 10 bees per cage, each with 1 g of commercially purchased pollen (Betterbee, Greenwich, NY) infused with pesticide treatments described below. Pollen was kept in the cages to provide continuous pollen intake for the bees throughout the pre-EAG testing period. Bees within the natural nurse bee window, seven to nine days old, were randomly selected for EAG testing. Thus, pollen feeding (or pesticide exposure) spanned at least seven days and up to nine days. Each cage also contained two tube feeders, one with 2 mL of sucrose water (50% w/v) and another with 2 mL of deionized water. To ensure comparability with the results of the queen-rearing assay conducted by Ricke et al.^36^, we used the same batch of treated pollen from the study. This approach ensured consistency in pollen composition and minimized potential variations in nutrition, associated phytochemicals, and technical factors during preparation and enabled a valid cross-comparison of the results of the two studies. Four pollen treatments were prepared by mixing homogenized commercial bee-collected pollen (BetterBee, Greenwich, NY) with formulated adjuvant, insecticide, and fungicide products at the subsequent concentrations: (1) 0.8% (w/w) Dyne-Amic, (2) Altacor (targeting 40 ppm of the active ingredient chlorantraniliprole) plus Tilt (targeting 90 ppm of active ingredient propiconazole), (3) 0.8% Dyne-Amic mixed Altacor and Tilt at the same targeted active ingredient concentrations, and (4) distilled water as the negative control. Concentrations were chosen based on the maximum field application rates for each product in almonds (see Supplementary Table 1 in Ricke et al.^36^) to simulate field exposure following a single pesticide application event. Each cage was randomly assigned one type of treated pollen. Based on the daily pollen consumption of 3.4 to 4.3 mg per bee reported by Crailsheim et al.^99^, we estimated that each bee ingested approximately 0.31–0.39 μg, 0.14–0.17 μg, and 27.20–34.40 μg per day of propiconazole, chlorantraniliprole, and Dyne-Amic, respectively. In the study by Ricke et al.^36^, nurse bees had average residues of 0.08 μg propiconazole and 0.01 μg chlorantraniliprole per bee per day, using the same treated pollen for bee feeding as in our study. This pollen sharing provides a reliable reference basis for hypothesized residue levels in our study bees^36^. Surprisingly, the reported pesticide residues in bees are lower than the estimates. This difference may be due to differences in pollen consumption between studies. In addition, field conditions may contribute to this difference, as bees in the study Ricke et al.^36^ had the opportunity to defecate, which likely reduced toxicant accumulation, whereas our estimate takes into account all possible toxicants ingested. Tubes of sucrose-water and water were replaced every two days. Bees were kept in a dark incubator (34 ± 1 °C, 50 ± 10% RH) before the EAG assay.

### Antenna preparation for electroantennogram (EAG) recording

Due to time, equipment, and environmental constraints, our capacity was limited to recording only 6 to 12 antennae per day. Therefore, to ensure a consistent supply of bees within the age range for sampling and conducting the EAG assay, day-old bees were arranged in cages with treated pollen, with one replicate performed per day. Each replicate covered a two- to three-day sampling period. This setup was repeated every two or three days over the course of six weeks. Bees within the nurse bee time window from a natural hive^100^, i.e., seven-, eight-, or nine-day old adults, were randomly sampled in cages. The sampled bee group alternated between the water control group and the other chemical groups.

After anesthetizing a bee on ice, one antenna was randomly selected and carefully removed by cutting at the base of the antennal scape using a micro-scissor. The proximal end of the antenna was inserted into a glass capillary filled with bee saline (130 mM NaCl, 6 mM KCl, 4 mM MgCl_2_, 5 mM CaCl_2_, 160 mM sucrose, 25 mM glucose, 10 mM HEPES, pH 6.7, 500 mOsmol)^101^. A droplet of biomedical-grade adhesive (KWIK-SIL, World Precision Instruments) was immediately applied to seal the opening between the capillary wall and the antenna to prevent saline evaporation and minimize electrical potential drift during the EAG recording. The glass capillary, containing the mounted antenna was fixed onto a micromanipulator (MM-3, Narishige International USA, INC.). The tip of the flagellum was also cut off to create a wound opening, which was then immersed and sealed in a separate glass capillary filled with the bee saline. This capillary was also mounted on another micromanipulator. The positioning of the antenna allowed for exposure to an odorant-carrying air stream by the antenna, except for the 10th segment of flagellum, distal part of 9th segment of flagellum, and proximal part of scapus, which were blocked from stimulus by saline and/or adhesive. Thus, our study may miss the contribution of these antenna segments to odor perception because their receptors may not receive the odorant stimulus tested. Our goal was to include as many segments as possible, and the sacrifice of some segments for the connection of electrical probes for recording was a necessary compromise that may, however, result in underestimating their role in odor perception. Mounting and sealing procedures were performed under a surgical microscope at 32 to 45 × magnification to ensure accurate and consistent preparation of the antennae for EAG recordings.

Two silver chloride-coated silver electrodes were immersed in the saline within the glass capillaries to pick up the electrophysiological signals from the antenna. The received electrical signal was then measured by a differential amplifier equipped with a headstage (Model 3000, A-M Systems). Subsequently, the signal underwent filtering using a notch filter and a 100 Hz low-pass filter, and was amplified by a factor of 1,000. A 16-bit multifunction data acquisition (DAQ) device (PCI-6036E, National Instrument) was utilized to digitalize the amplified signal at a sampling rate of 1000 samples per second. All data acquisition and processing were controlled by a custom LabVIEW program (ver. 2018, National Instrument) developed specifically for this purpose.

### Odor stimulus preparation

To elicit the olfactory response of the bee antenna, three types of stimuli were used:

1. A 10-fatty acid ester-blend was prepared to mimic a semi-volatile brood-emitted ester pheromone (BEP)^39,40^ based on the study of Pankiw et al.^38^. The composition of the blend included methyl oleate (24.95%; 311111, Sigma-Aldrich), methyl linolenate (21%; 62200, Supelco), methyl stearate (17%; S0080, TCI America), ethyl linolenate (13%; 10008199, Cayman Chemical), ethyl oleate (8%; 268011, Sigma-Aldrich), ethyl stearate (7%; S0079, TCI America), methyl palmitate (3%; P0750, Sigma), ethyl palmitate (3%; P9009, Sigma-Aldrich), methyl linoleate (2%; 62280, Supelco), ethyl linoleate (1%; L0055, TCI America), and the preservative, tertiary-butylhydroquinone (0.05%; 112941, Sigma-Aldrich). Solutions of this synthetic BEP in mineral oil (M8410, Sigma-Aldrich) were prepared in concentrations at intervals on a logarithm scale (0%, 5%, 10%, 20%, 40%, 100%) to obtain dose-dependent responses. These solutions were stored at -20 °C until used in the assays.
2. The terpene β-ocimene (CAS No. 13877–91-3; W353901, Sigma-Aldrich), comprising both (E)-β-ocimene and (Z)-β-ocimene, was employed to mimic another highly volatile larval signal. (E)-β-ocimene is known to act as a brood pheromone from larvae that has both releaser and primer effects^41^. Additionally, Noël et al.^41^ suggested that nurse bees may be capable of detecting and utilizing both β-ocimene enantiomers, because (Z)-β-ocimene is also abundantly released by older larvae and pupae within the brood.
3. The ketone 2-heptanone (2185, Eastman Kodak Company) was used to imitate a volatile worker-emitted alarm pheromone for defense^42^.

To prepare the odor stimuli, 500 µL of the solutions of BEP, β-ocimene, and 2-heptanone were pipetted into uncapped 4-mL glass vials, which were then placed into 20-mL headspace gas sampling vials. The chemicals were allowed to vaporize and emit odors into the headspace of the gas sampling vials during the assay (Fig. 2A). The 4-mL glass vials containing the chemicals were changed daily to ensure consistent odor presentation.

### Delivery of odor stimuli

The odor stimuli were delivered to the tested antenna using an olfactometer utilizing a round glass tube with a 1-cm opening diameter, which was positioned 1 cm away from the antenna. A stimulus controller (CS-55, Syntech), incorporating a charcoal filter, two air pumps, and a solenoid valve, supplied clean air as the airstream source and controlled the rate and ratio of the diverted flows.

The delivered airstream, flowing at a constant rate of approximately 15 cm/s, was used to allow the bristle sensilla to adapt. Six-sevenths of the airstream’s volume flow consisted of a continuous stream of air moisturized through a heated humidifier, while the remaining one-seventh alternated between a stimulus air pulse and a complementary air pulse. During odor stimulation, the stimulus air pulse was generated and directed through a set of solenoid valves (three-way, normally closed, VT317V, SMC Corporation). To ensure consistent volume flows for the stimulus and complementary air pulses across all paths, we regulated diverted flows with the stimulus air controller and used airflow control valves at the outlets of solenoid valves connecting to sampling vials. The stimulus pulse was passed through one of the headspace gas sampling vials containing the stimulus odor, blowing it out into the airstream.

To overcome the limited volatilization of the BEP stimuli at room temperature, we used a heating technique that has previously been shown to be effective in capturing responses to low-volatility odorants^102^. Six headspace gas sampling vials containing the synthetic BEP and mineral oil were heated in an oven at 55 °C to facilitate their volatilization^102^. The headspace gas sampling vials containing volatilized β-ocimene and 2-heptanone were kept at room temperature to accommodate their high volatility. Heating β-ocimene and 2-heptanone might lead to excessive concentrations of volatiles, potentially saturating the antenna receptors.

The overall electroantennogram recording and stimulation apparatus set-up is shown in Fig. 2A. The tubes carrying the humidified continuous air, the stimulating air pulse, and the complementary air pulse were combined in a glass mixing chamber within the oven. Airflows were mixed in the chamber before delivery to the tested antenna. The temperature of the final delivered air stream was maintained at 30 ± 2 °C, monitored by a type K bead wire thermocouple with a thermometer (800077 & 800004, Sper Scientific).

Each tested antenna underwent a minimum of six series of eight odor stimuli, with a 17-s interval between each stimulus. The duration of each air pulse was 200 ms (Fig. 2B). The overall response delay, including the mechanical delay of the odor delivery system and the delay of the EAG response, was approximately 160 ms.

### EAG recording

EAG measures and records the potential drop across the antenna resulting from the activation of odorant receptors and the subsequent opening of ion channels. The response amplitude was calculated by determining the peak potential, which is the most negative potential recorded within 240 ms after the onset of the EAG response (Fig. 2B). The baseline potential was obtained by averaging the potentials recorded during the 100 ms period before the onset of the electrophysiological response. The response amplitude was then calculated by subtracting the baseline potential from the peak potential. To reduce the impact of background noise, two replicated stimulative air pulses of the same odor were administered with a 17-s interval, and the responses to these replicated stimuli were averaged.

The recording of responses from six series of stimuli took approximately 30 min. Only the data from antennae that continued to respond to 2-heptanone until the sixth round of stimuli were included in the final analysis. The final analysis encompassed the responses recorded from the antennae of 106 worker bees.

### Data analysis

A total of 5,088 responses were recorded from 106 bees in two hives, with 23–30 bees in each treatment group (Table S1). Each bee antenna underwent exposure to eight odor stimuli in six replicates, resulting in 48 responses per antenna. The first two series of stimuli were used for flushing and priming the odor delivery system and were not included in the final analysis ((6 - 2 replicate series) × 8 odor stimuli × 106 bees = 3392 responses). Two accidental airflow interference events resulted in the exclusion of two sets of stimulation repetitions (3392 - 8 × 2 = 3376 responses). Additionally, seven negative amplitude responses, which occasionally occurred in response to mineral oil odors and were likely due to noise, were excluded from the final analysis as they appeared unrealistic (3376 - 7 = 3369 responses). The final analysis included 3,369 EAG responses (Table S2).

The Generalized Estimating Equations (GEE) method, as described by Hardin and Hilbe^103^, was employed for analyzing the EAG data. Individual bees were treated as subjects, and the recorded response to each chemical at each time point served as the within-subject variable for the analysis of repeated measures. An unstructured correlation matrix was used under the model-based estimator. The dependent variable was the amplitude of the EAG response, while the fungicide treatments were considered fixed factors, with the water control group designated as the reference category. The hives and the series stimuli order were adjusted as covariates in all analyses. The models were fitted using IBM SPSS Statistics v. 24 (SPSS Inc., Chicago, IL, USA).

### Supplementary Information

Supplementary Information.

## Data Availability

The datasets and custom programs generated during this study are available from the corresponding author on reasonable request.

## References

[1] Siviter H (2021). Agrochemicals interact synergistically to increase bee mortality. Nature.

[2] Tosi S, Sfeir C, Carnesecchi E, vanEngelsdorp D, Chauzat M-P (2022). Lethal, sublethal, and combined effects of pesticides on bees: A meta-analysis and new risk assessment tools. Sci. Total Environ..

[3] Pervez M, Manzoor F (2023). Honey bee losses and pesticides threat: an Asian perspective. J. Apic. Res..

[4] Zhu, W., Schmehl, D. R., Mullin, C. A. & Frazier, J. L. Four common pesticides, their mixtures and a formulation solvent in the hive environment have high oral toxicity to honey bee larvae. *PLoS ONE* **9**, e77547, 10.1371/journal.pone.0077547 (2014). 10.1371/journal.pone.0077547 PMC3885384 24416121

[5] Mullin CA, Chen J, Fine JD, Frazier MT, Frazier JL (2015). The formulation makes the honey bee poison. Pestic. Biochem. Physiol..

[6] Straw EA, Carpentier EN, Brown MJF (2021). Roundup causes high levels of mortality following contact exposure in bumble bees. J. Appl. Ecol..

[7] Motta, E. V. S. & Moran, N. A. The effects of glyphosate, pure or in herbicide formulation, on bumble bees and their gut microbial communities. *Sci. Total Environ.*, 10.1016/j.scitotenv.2023.162102 (2023). 10.1016/j.scitotenv.2023.162102 PMC11050743 36764553

[8] Mesnage R, Antoniou MN (2018). Ignoring adjuvant toxicity falsifies the safety profile of commercial pesticides. Front. Public Health.

[9] Chen J, Mullin CA (2013). Quantitative determination of trisiloxane surfactants in beehive environments based on liquid chromatography coupled to mass spectrometry. Environ. Sci. Technol..

[10] Chen J, Mullin CA (2015). Characterization of trisiloxane surfactants from agrochemical adjuvants and pollinator-related matrices using liquid chromatography coupled to mass spectrometry. J. Agric. Food Chem..

[11] Collins JK, Jackson JM (2022). Application of a screening-level pollinator risk assessment framework to trisiloxane polyether surfactants. Environ. Toxicol. Chem..

[12] Cowles RS, Cowles EA, McDermott AM, Ramoutar D (2000). Inert formulation ingredients with activity: Toxicity of trisiloxane surfactant solutions to twospotted spider mites (Acari: Tetranychidae). J. Econ. Entomol..

[13] Tipping C, Bikoba V, Chander GJ, Mitcham EJ (2003). Efficacy of Silwet L-77 against several arthropod pests of table grape. J. Econ. Entomol..

[14] A. Cloyd, R. Effects of pesticides and adjuvants on the honey bee, *Apis mellifera*: An updated bibliographic review. in *Modern Beekeeping - Bases for Sustainable Production* (ed Ramón Eduardo Rebolledo Ranz) Ch. Chapter 1, (IntechOpen, 2020).

[15] Mullin CA (2015). Effects of ‘inactive’ ingredients on bees. Curr. Opin. Insect Sci..

[16] Nagy K (2020). Systematic review of comparative studies assessing the toxicity of pesticide active ingredients and their product formulations. Environ. Res..

[17] Straw EA, Thompson LJ, Leadbeater E, Brown MJF (2022). ‘Inert’ ingredients are understudied, potentially dangerous to bees and deserve more research attention. Proc. R. Soc. B.

[18] Ciarlo TJ, Mullin CA, Frazier JL, Schmehl DR (2012). Learning impairment in honey bees caused by agricultural spray adjuvants. PLoS ONE.

[19] Goodwin RM, McBrydie HM (2000). Effect of surfactants on honey bees. N. Z. Plant Prot..

[20] Wernecke A, Eckert JH, Forster R, Kurlemann N, Odemer R (2022). Inert agricultural spray adjuvants may increase the adverse effects of selected insecticides on honey bees (*Apis mellifera* L.) under laboratory conditions. J. Plant Dis. Prot..

[21] Walker EK, Brock GN, Arvidson RS, Johnson RM (2022). Acute toxicity of fungicide–insecticide–adjuvant combinations applied to almonds during bloom on adult honey bees. Environ. Toxicol. Chem..

[22] Fine JD, Cox-Foster DL, Mullin CA (2017). An inert pesticide adjuvant synergizes viral pathogenicity and mortality in honey bee larvae. Sci. Rep..

[23] Slessor KN, Winston ML, Conte Y (2005). Pheromone communication in the honeybee (*Apis mellifera* L.). J. Chem. Ecol..

[24] Bortolotti, L. & Costa, C. Chemical communication in the honey bee society. in *Neurobiology of Chemical Communication Frontiers in Neuroscience* (ed C. Mucignat-Caretta) Ch. 5, 147–210 (CRC Press, 2014). 24830041

[25] Paoli M, Galizia GC (2021). Olfactory coding in honeybees. Cell Tissue Res..

[26] Müller C (2018). Impacts of sublethal insecticide exposure on insects — Facts and knowledge gaps. Basic Appl. Ecol..

[27] Tricoire-Leignel H, Thany SH, Gadenne C, Anton S (2012). Pest insect olfaction in an insecticide-contaminated environment: info-disruption or hormesis effect. Front. Physiol..

[28] Renou M, Anton S (2020). Insect olfactory communication in a complex and changing world. Curr. Opin. Insect Sci..

[29] Chakrabarti P (2015). Field populations of native Indian honey bees from pesticide intensive agricultural landscape show signs of impaired olfaction. Sci. Rep..

[30] Siviter H, Koricheva J, Brown MJF, Leadbeater E (2018). Quantifying the impact of pesticides on learning and memory in bees. J. Appl. Ecol..

[31] Desneux N, Decourtye A, Delpuech J-M (2007). The sublethal effects of pesticides on beneficial arthropods. Annu. Rev. Entomol..

[32] Alkassab AT, Kirchner WH (2017). Sublethal exposure to neonicotinoids and related side effects on insect pollinators: Honeybees, bumblebees, and solitary bees. J. Plant Dis. Prot..

[33] Flottum, K. *Catch the buzz: Huge bee kill in almonds* https://www.beeculture.com/huge-bee-kill-in-almonds/ (2014).

[34] Wade A, Lin C-H, Kurkul C, Regan E, Johnson RM (2019). Combined toxicity of insecticides and fungicides applied to California almond orchards to honey bee larvae and adults. Insects.

[35] Liao L-H (2020). Increase in longevity and amelioration of pesticide toxicity by natural levels of dietary phytochemicals in the honey bee, *Apis mellifera*. PLoS ONE.

[36] Ricke DF, Lin C-H, Johnson RM (2021). Pollen treated with a combination of agrochemicals commonly applied during almond bloom reduces the emergence rate and longevity of honey bee (Hymenoptera: Apidae) queens. J. Insect Sci..

[37] Berenbaum MR, Liao L-H (2019). Honey bees and environmental stress: Toxicologic pathology of a superorganism. Toxicol. Pathol..

[38] Pankiw T, Birmingham AL, Lafontaine JP, Avelino N, Borden JH (2011). Stabilized synthetic brood pheromone delivered in a slow-release device enhances foraging and population size of honey bee, *Apis mellifera*, colonies. J. Apic. Res..

[39] Le Conte Y, Arnold G, Trouiller J, Masson C, Chappe B (1990). Identification of a brood pheromone in honeybees. Naturwissenschaften.

[40] Le Conte Y, Mohammedi A, Robinson GE (2001). Primer effects of a brood pheromone on honeybee behavioural development. Proc. R. Soc. Lond. Ser. B Biol. Sci..

[41] Noël A (2023). Detailed chemical analysis of honey bee (*Apis mellifera*) worker brood volatile profile from egg to emergence. PLoS ONE.

[42] Shearer DA, Boch R (1965). 2-Heptanone in the mandibular gland secretion of the honey-bee. Nature.

[43] Breed MD, Guzmán-Novoa E, Hunt GJ (2004). Defensive behavior of honey bees: organization, genetics, and comparisons with other bees. Annu. Rev. Entomol..

[44] Ohlinger BD (2022). Honey bees (Hymenoptera: Apidae) decrease foraging but not recruitment after neonicotinoid exposure. J. Insect Sci..

[45] Jiang X (2018). The effect of neonicotinoid insecticide and fungicide on sugar responsiveness and orientation behavior of honey bee (*Apis mellifera*) in semi-field conditions. Insects.

[46] Williamson SM, Wright GA (2013). Exposure to multiple cholinergic pesticides impairs olfactory learning and memory in honeybees. J. Exp. Biol..

[47] Decourtye A (2005). Comparative sublethal toxicity of nine pesticides on olfactory learning performances of the honeybee *Apis mellifera*. Arch. Environ. Contam. Toxicol..

[48] Aliouane Y (2009). Subchronic exposure of honeybees to sublethal doses of pesticides: effects on behavior. Environ. Toxicol. Chem..

[49] Bell HC, Montgomery CN, Benavides JE, Nieh JC (2020). Effects of *Nosema ceranae* (Dissociodihaplophasida: Nosematidae) and flupyradifurone on olfactory learning in honey bees, *Apis mellifera* (Hymenoptera: Apidae). J. Insect Sci..

[50] Decourtye A, Devillers J, Cluzeau S, Charreton M, Pham-Delègue M-H (2004). Effects of imidacloprid and deltamethrin on associative learning in honeybees under semi-field and laboratory conditions. Ecotoxicol. Environ. Saf..

[51] Decourtye A (2004). Imidacloprid impairs memory and brain metabolism in the honeybee (*Apis mellifera* L.). Pestic. Biochem. Physiol..

[52] Guez D, Belzunces LP, Maleszka R (2003). Effects of imidacloprid metabolites on habituation in honeybees suggest the existence of two subtypes of nicotinic receptors differentially expressed during adult development. Pharmacol. Biochem. Behav..

[53] Guez D, Suchail S, Gauthier M, Maleszka R, Belzunces LP (2001). Contrasting effects of imidacloprid on habituation in 7- and 8-day-old honeybees (*Apis mellifera*). Neurobiol. Learn. Mem..

[54] Goñalons, C. M., Farina, W. M. Impaired associative learning after chronic exposure to pesticides in young adult honey bees. *J. Exp. Biol.* **221**, jeb176644, 10.1242/jeb.176644 (2018). 10.1242/jeb.176644 29643175

[55] Ke L, Chen X, Dai P, Liu Y-J (2023). Chronic larval exposure to thiacloprid impairs honeybee antennal selectivity, learning and memory performances. Front. Physiol..

[56] DesJardins NS (2021). A common fungicide, Pristine®, impairs olfactory associative learning performance in honey bees (*Apis mellifera*). Environ. Pollut..

[57] Lim S, Yunusbaev U, Ilyasov R, Lee HS, Kwon HW (2020). Abdominal contact of fluvalinate induces olfactory deficit in *Apis mellifera*. Pestic. Biochem. Physiol..

[58] Kadala A, Charreton M, Jakob I, Conte YL, Collet C (2011). A use-dependent sodium current modification induced by type I pyrethroid insecticides in honeybee antennal olfactory receptor neurons. Neurotoxicology.

[59] Palmer MJ (2013). Cholinergic pesticides cause mushroom body neuronal inactivation in honeybees. Nat. Commun..

[60] Gao J (2023). Acute oral toxicity, apoptosis, and immune response in nurse bees (*Apis mellifera*) induced by flupyradifurone. Front. Physiol..

[61] El Hassani AK, Giurfa M, Gauthier M, Armengaud C (2008). Inhibitory neurotransmission and olfactory memory in honeybees. Neurobiol. Learn. Mem..

[62] Aguiar JMRBV, Nocelli RCF, Giurfa M, Nascimento FS (2023). Neonicotinoid effects on tropical bees: Imidacloprid impairs innate appetitive responsiveness, learning and memory in the stingless bee *Melipona quadrifasciata*. Sci. Total Environ..

[63] David NF, Henry TJ, Sprayberry JDH (2022). Odor-pollution from fungicides disrupts learning and recognition of a common floral scent in bumblebees (*Bombus impatiens*). Front. Ecol. Evol..

[64] Thompson LJ, Stout JC, Stanley DA (2023). Contrasting effects of fungicide and herbicide active ingredients and their formulations on bumblebee learning and behaviour. J. Exp. Biol..

[65] Fisher, A., DeGrandi-Hoffman, G., Liao, L.-H., Tadei, R. & Harrison, J. F. The challenge of balancing fungicide use and pollinator health. in *Environmental Threats to Pollinator Health and Fitness Advances in Insect Physiology* (ed Jon F. Harrison) (Academic Press, 2023).

[66] Schuhmann A, Schmid AP, Manzer S, Schulte J, Scheiner R (2022). Interaction of insecticides and fungicides in bees. Front. Insect Sci..

[67] Casida JE, Durkin KA (2013). Neuroactive insecticides: targets, selectivity, resistance, and secondary effects. Annu. Rev. Entomol..

[68] Li A (2023). Thiacloprid impairs honeybee worker learning and memory with inducing neuronal apoptosis and downregulating memory-related genes. Sci. Total Environ..

[69] Xu X (2022). Neonicotinoids: mechanisms of systemic toxicity based on oxidative stress-mitochondrial damage. Arch. Toxicol..

[70] Jacob VEJM (2018). Current source density analysis of electroantennogram recordings: A tool for mapping the olfactory response in an insect antenna. Front. Cell. Neurosci..

[71] Kohlmeier P, Billeter JC (2023). Genetic mechanisms modulating behaviour through plastic chemosensory responses in insects. Mol. Ecol..

[72] Rihani K, Ferveur J-F, Briand L (2021). The 40-year mystery of insect odorant-binding proteins. Biomolecules.

[73] Larter, N. K., Sun, J. S., Carlson, J. R. Organization and function of *Drosophila* odorant binding proteins. *eLife* **5**, e20242, 10.7554/elife.20242 (2016). 10.7554/eLife.20242 PMC5127637 27845621

[74] Schmidt HR, Benton R (2020). Molecular mechanisms of olfactory detection in insects: beyond receptors. Open Biol..

[75] Mayack, B. K. Modeling disruption of *Apis mellifera* (honey bee) odorant‐binding protein function with high‐affinity binders. *J. Mol. Recognit.* **36**, e3008, 10.1002/jmr.3008 (2023). 10.1002/jmr.3008 36792370

[76] Colgan TJ (2019). Caste- and pesticide-specific effects of neonicotinoid pesticide exposure on gene expression in bumblebees. Mol. Ecol..

[77] Li H (2015). Neonicotinoid insecticide interact with honeybee odorant-binding protein: Implication for olfactory dysfunction. Int. J. Biol. Macromol..

[78] Shi T-F, Wang Y-F, Liu F, Qi L, Yu L-S (2017). Sublethal effects of the neonicotinoid insecticide thiamethoxam on the transcriptome of the honey bees (Hymenoptera: Apidae). J. Econ. Entomol..

[79] Jayaram CS, Chauhan N, Dolma SK, Reddy SGE (2020). Deformation of appendages, antennal segments and sensilla of aphid (*Aphis craccivora* Koch) treated with *Tagetes minuta* oil: a scanning electron microscopy study. Toxin Rev..

[80] Agwunobi DO, Pei T, Wang K, Yu Z, Liu J (2020). Effects of the essential oil from *Cymbopogon citratus* on mortality and morphology of the tick *Haemaphysalis longicornis* (Acari: Ixodidae). Exp. Appl. Acarol..

[81] Jibrin MO, Liu Q, Jones JB, Zhang S (2021). Surfactants in plant disease management: A brief review and case studies. Plant Pathol..

[82] Mullin CA, Fine JD, Reynolds RD, Frazier MT (2016). Toxicological risks of agrochemical spray adjuvants: Organosilicone surfactants may not be safe. Front. Public Health.

[83] Deisig N, Giurfa M, Lachnit H, Sandoz JC (2006). Neural representation of olfactory mixtures in the honeybee antennal lobe. Eur. J. Neurosci..

[84] Deisig N, Giurfa M, Sandoz JC (2010). Antennal lobe processing increases separability of odor mixture representations in the honeybee. J. Neurophysiol..

[85] Kuebler LS, Olsson SB, Weniger R, Hansson BS (2011). Neuronal processing of complex mixtures establishes a unique odor representation in the moth antennal lobe. Front. Neural Circ..

[86] Kuebler LS, Schubert M, Kárpáti Z, Hansson BS, Olsson SB (2012). Antennal lobe processing correlates to moth olfactory behavior. J. Neurosci..

[87] Chaffiol A (2012). Plant odour stimuli reshape pheromonal representation in neurons of the antennal lobe macroglomerular complex of a male moth. J. Exp. Biol..

[88] Rabhi, K. K. *et al.* Unexpected effects of low doses of a neonicotinoid insecticide on behavioral responses to sex pheromone in a pest insect. *PLoS ONE* **9**, e114411, 10.1371/journal.pone.0114411 (2014). 10.1371/journal.pone.0114411 PMC4269385 25517118

[89] Rabhi KK (2016). Low doses of a neonicotinoid insecticide modify pheromone response thresholds of central but not peripheral olfactory neurons in a pest insect. Proc. R. Soc. B.

[90] Lucas P, Renou M (1992). Electrophysiological study of the effects of deltamethrin, bioresmethrin, and DDT on the activity of pheromone receptor neurones in two moth species. Pestic. Biochem. Physiol..

[91] Ai S (2022). Insect-microorganism interaction has implicates on insect olfactory systems. Insects.

[92] Tumkaya, T. *et al.* Most primary olfactory neurons have individually neutral effects on behavior. *eLife* **11**, e71238, 10.7554/elife.71238 (2022). 10.7554/eLife.71238 PMC8806191 35044905

[93] Wilson RI (2008). Neural and behavioral mechanisms of olfactory perception. Curr. Opin. Neurobiol..

[94] Olsson, S. B. & Hansson, B. S. Electroantennogram and single sensillum recording in Insect antennae. in *Pheromone Signaling* Vol. 1068 *Methods in Molecular Biology* (ed K. Touhara) 157–177 (Humana Press, 2013). 10.1007/978-1-62703-619-1_11 24014360

[95] Pawson SM (2020). Light-weight portable electroantennography device as a future field-based tool for applied chemical ccology. J. Chem. Ecol..

[96] Groot PD (2008). Electrophysiological response and attraction of emerald ash borer to green leaf volatiles (GLVs) emitted by host foliage. J. Chem. Ecol..

[97] Lalouette L (2016). Unexpected effects of sublethal doses of insecticide on the peripheral olfactory response and sexual behavior in a pest insect. Environ. Sci. Pollut. Res..

[98] Shpigler HY, Robinson GE (2015). Laboratory assay of brood care for quantitative analyses of individual differences in honey bee (*Apis mellifera*) affiliative behavior. PLoS ONE.

[99] Crailsheim K (1992). Pollen consumption and utilization in worker honeybees (*Apis mellifera carnica*): Dependence on individual age and function. J. Insect Physiol..

[100] Seeley TD (1982). Adaptive significance of the age polyethism schedule in honeybee colonies. Behav. Ecol. Sociobiol..

[101] Faber T, Menzel R (2001). Visualizing mushroom body response to a conditioned odor in honeybees. Naturwissenschaften.

[102] Carcaud J, Giurfa M, Sandoz J-C (2015). Differential combinatorial coding of pheromones in two olfactory subsystems of the honey bee brain. J. Neurosci..

[103] Hardin, J. W. & Hilbe, J. M. *Generalized Estimating Equations*. 2nd edn, 277 (CRC Press, LLC, 2013).

