# Supplementary material for: Effects of pesticide-adjuvant combinations used in almond orchards on olfactory responses to social signals in honey bees (Apis mellifera)
Source: Sci Rep. 2023 Sep 20;13:15577. doi: 10.1038/s41598-023-41818-7 (PMC10511525; doi:10.1038/s41598-023-41818-7)
Supplement: Supplementary file 1 — Supplementary Information. [file 41598_2023_41818_MOESM1_ESM.docx]

Table S1. Number of honey bees reared on pesticide-treated pollen tested in this study for responses to pheromonal stimuli

|  | Hive 1 | Hive 2 | sub total |
| --- | --- | --- | --- |
| Water | 10 | 20 | 30 |
| Dyne-Amic | 12 | 14 | 26 |
| Altacor + Tilt | 10 | 13 | 23 |
| Dyne-Amic + Altacor + Tilt | 10 | 17 | 27 |
| sub total | 42 | 64 | 106 |

Table S2. The number of EAG responses were included in the final analysis

|  | Mineral oil | 5%BEP ^a^ | 10%BEP | 20%BEP | 40%BEP | 100% BEP | β-Ocimene | 2-heptanone | sub total |
| --- | --- | --- | --- | --- | --- | --- | --- | --- | --- |
| Water | 119 | 119 | 119 | 119 | 119 | 119 | 119 | 119 | 952 |
| Dyne-Amic | 103 | 104 | 104 | 104 | 104 | 104 | 104 | 104 | 831 |
| Altacor + Tilt | 89 | 92 | 92 | 92 | 92 | 92 | 92 | 92 | 733 |
| Dyne-Amic + Altacor + Tilt | 104 | 107 | 107 | 107 | 107 | 107 | 107 | 107 | 853 |
| sub total | 415 | 422 | 422 | 422 | 422 | 422 | 422 | 422 | 3369 |

^a.^ BEP: mixed 10-in-1 brood-emitted ester pheromone
